# Characterization of Degenerative Mitral Valve Disease: Differences between Fibroelastic Deficiency and Barlow’s Disease

**DOI:** 10.3390/jcdd8020023

**Published:** 2021-02-22

**Authors:** Aniek L. van Wijngaarden, Boudewijn P. T. Kruithof, Tommaso Vinella, Daniela Q. C. M. Barge-Schaapveld, Nina Ajmone Marsan

**Affiliations:** 1 Department of Cardiology, Leiden University Medical Center, Albinusdreef 2, 2333 ZA Leiden, The Netherlands; a.l.van_wijngaarden@lumc.nl (A.L.v.W.); B.P.T.Kruithof@lumc.nl (B.P.T.K.); 2 Faculty of Medicine, University of Southampton, Southampton SO17 1BJ, UK; tv3u17@soton.ac.uk; 3 Department of Clinical Genetics, Leiden University Medical Center, 2333 ZA Leiden, The Netherlands; D.Q.C.M.Barge-Schaapveld@lumc.nl

**Keywords:** mitral valve prolapse, imaging, mitral valve surgery

## Abstract

Degenerative mitral valve disease causing mitral valve prolapse is the most common cause of primary mitral regurgitation, with two distinct phenotypes generally recognized with some major differences, i.e., fibroelastic deficiency (FED) and Barlow’s disease. The aim of this review was to describe the main histological, clinical and echocardiographic features of patients with FED and Barlow’s disease, highlighting the differences in diagnosis, risk stratification and patient management, but also the still significant gaps in understanding the exact pathophysiology of these two phenotypes.

## 1. Introduction

Mitral regurgitation (MR) is the second most common valvular disorder worldwide [1] and can be divided into primary (due to intrinsic lesions of the mitral valve apparatus) and secondary (due to a disease of the left ventricle) MR. The Carpentier classification, which is based on the mobility of the mitral valve leaflets, further divides primary MR in three different types: (1) Type I, when the motion of the mitral valve leaflets is normal but MR is due to a leaflet perforation or cleft; (2) Type II, when the mitral valve leaflets have excessive mobility, as in the case of leaflet prolapse or flail; (3) Type IIIa, when the mitral valve leaflets have restricted mobility in systole and diastole, as in the case of leaflet thickening and/or calcification [2].

Degenerative mitral valve disease causing mitral valve prolapse (Carpentier type II) is the most common cause of primary MR, with two distinct phenotypes generally recognized with some major differences, i.e., fibroelastic deficiency (FED) and Barlow’s disease [3]. However, patients may share characteristics of both phenotypes and a clear distinction cannot always be made. The aim of this review is to describe the main histological, clinical and echocardiographic features of patients with FED and Barlow’s disease, highlighting the differences in diagnosis, risk stratification and patient management, but also the still significant gaps in understanding the exact pathophysiology of these two phenotypes.

## 2. History

The occurrence of a systolic murmur in combination with a midsystolic click was described since 1887. However, it was not until the 1960s that Barlow et al. [4] demonstrated, using left ventricular (LV) cineangiography, that these phenomena were caused by MR. Initially, the authors hypothesized that rheumatic disease was the underlying mechanism of MR. Criley et al. [5] corrected this hypothesis, revealing that the regurgitation was due to excessive posterior leaflet motion into the left atrium during systole, and called this abnormality mitral valve prolapse. The macroscopic characteristics of the Barlow’s valve were described using histological examination, including diffuse thickening of the leaflets and elongation of the chordae, and myxomatous degeneration was identified as the underlying mechanism, instead of rheumatic disease [6]. Carpentier et al. [7] confirmed these aspects of the Barlow’s disease by surgical exploration, but also identified another type of mitral valve prolapse without redundant leaflet tissue, instead, mainly due to chordal rupture, which was called FED.

## 3. Pathophysiological Mechanism

The mitral valve has an anterior and a posterior leaflet, which are both divided into three segments (P1, P2 and P3 scallops for the posterior leaflet and A1, A2 and A3 scallops for the anterior leaflet). Normal mitral valve tissue consists of three layers, namely, the atrialis, the spongiosa and the fibrosa. The atrialis, on the atrial side, is rich in elastic fibers, providing elasticity to the valve. The spongiosa, the middle layer, is principally made of glycosaminoglycans and proteoglycans, supplying flexibility to the valve, absorbing vibrations and cushioning the valve from the shock of closure. The fibrosa, on the ventricular side, is the thickest part of the leaflet and is rich in collagen fibers, providing tensile strength to the valve [8].

In Barlow’s disease, the organization of the three layers is disrupted. Collagen and elastin fibers are fragmented and the spongiosa layer expands due to the accumulation of proteoglycans, characteristic of the myxomatous degeneration, and infiltrates the fibrosa layer [9]. Thickening of the leaflet is therefore due to the myxomatous infiltration, but also the formation of fibrous tissue at the atrial and, to a lesser extent, the ventricular sides of the leaflets. Recently, it was shown that this fibrous tissue actually forms on top the original leaflet and was therefore called superimposed tissue [10,11]. In addition, it was shown that the superimposed tissue may have various compositions of the extracellular matrix [10] and that its formation is mainly induced by mechanical stress when simulating valve mechanics using an ex vivo model of a whole mouse heart [10]. These alterations of the leaflet structure cause also significant changes of the mechanical properties of the mitral valve, resulting in more extensible leaflets [12].

Mitral valve with FED, on the other hand, is characterized by leaflets thinning, which is thought to be due to impaired production of connective tissue, with deficiency of collagen, elastin and proteoglycans [9,13]. Focal chordae tendinea elongation or rupture causes prolapse of the mitral valve. However, thickened leaflet tissue is found at the level of the prolapsing segment, which displays myxomatous infiltration and superimposed tissue formation, as seen in Barlow’s disease.

**Figure 1:**
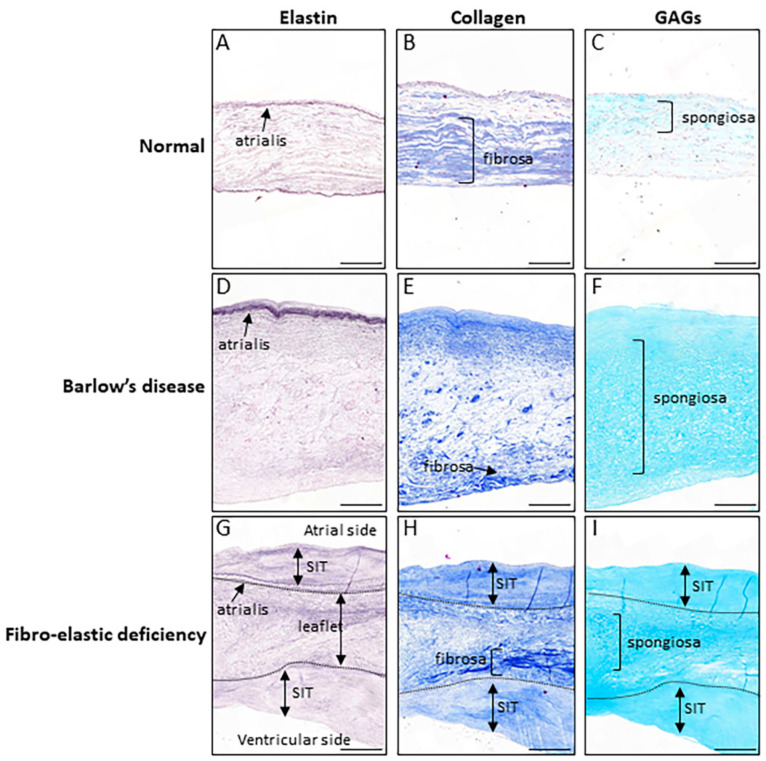
Morphological features of normal and degenerative mitral valve disease. Normal (**A**–**C**) and degenerative mitral valves (**D**–**I**) are stained for elastin (**A**,**D**,**G**; Weigert’s Resorcin Fuchsin), collagen (**B**,**E**,**H**; Masson’s Trichrome) and glycoaminoglycans (GAGs; **C**,**F**,**I**; Alcian blue). Degenerative mitral valves (**D**–**I**) are characterized by an abnormal organization of the extracellular matrix with disrupted elastin (**D**,**G**), diminished and loose collagen in the fibrosa layer (**E**) and expansion of the spongiosa layer (**F**,**I**). In addition, superimposed tissue (SIT) can be present at the atrial and ventricular sides of the original leaflet, contributing to increased thickness of the leaflet (**G**–**I**). Scalebar: 500 mm.

Whether FED and Barlow’s disease are part of a single degenerative mitral valve disease spectrum or two separate diseases is not completely clear. More recent studies using resected tissues obtained during surgery of mitral valve repair assessed the histological differences between FED and Barlow’s disease in detail [14]. In the resected tissue, both FED and Barlow’s disease leaflets displayed myxomatous degeneration with disruption of the three layers, with fragmented and disorganized collagen and elastin fibers and excessive production of proteoglycans (Figure 1) [15,16]. Collagen alterations and myxomatous infiltrations in the fibrosa were slightly more abundant in Barlow’s disease, while elastic fiber lesions appeared more present in FED [14]. A clear histological difference between FED and Barlow’s disease, however, was not found. These observations could suggest that FED and Barlow’s disease are part of the same degenerative mitral valve disease, which can affect the leaflets focally (as in FED) or in a more diffuse way (as in Barlow’s disease), as a spectrum of severity [17]. However, the resected tissue normally used for analysis is the excessive prolapsing part that, especially in FED, has a clearly distinct morphology compared with the rest of the leaflets and is the one exposed to regurgitation. As recently demonstrated, pathological remodeling of the leaflets can be induced by regurgitant flow even in initially healthy valves [10,18], supporting the observation that the prolapsing segments (the ones normally resected) show abnormalities possibly secondary to MR, which are shared by FED and Barlow’s disease, while the primary valvular defects are less distinguished [9,19]. Finally, the differences observed could be related to the time that the valve is exposed to regurgitation, which might be short in FED, due to acute chordae rupture, and longer in Barlow’s disease, where a certain degree of MR can be present for decades. Analyses of the thin part of the leaflet in FED and the nonprolapsing scallops of Barlow’s disease leaflets might provide further insight; however, these tissues are difficult to obtain because they are normally not resected.

Interestingly, analysis of resected chordae tendineae of FED and Barlow’s disease (sometimes removed even if not broken) presents more distinct patterns than the leaflets. FED is characterized by increased chordal thickness and stiffness due to focal deposition of elastin and collagen and increased expression of fibrocytes, whereas the chordae in Barlow’s disease seem to be less affected [9,14]. This might indicate that secondary induced pathological remodeling is less present at the chordal level. Therefore, chordae tendineae, although so far not largely studied, represent an attractive, available tissue to study the potential difference in etiology between FED and Barlow’s disease.

## 4. Genetic Background

Although the majority of mitral valve prolapse cases seem to be sporadic, a familial genetic basis was acknowledged. Delling et al. [20] reported in a community-based cohort (Framingham study) that parental mitral valve prolapse is associated with a higher prevalence of mitral valve prolapse in their offspring. A more recent study by Hiemstra et al. [21] showed a prevalence of family history of primary MR in 26% of Barlow’s disease patients and 8% of FED patients who underwent mitral valve surgery. These studies highlight the importance of specific familial anamnesis, and eventually screening, in all mitral valve prolapse patients, but particularly in Barlow’s disease, which clinically manifests in patients often in their fourth or fifth decade [22].

Three loci were recognized for autosomal dominant primary mitral valve prolapse, namely, chromosome 16p11.2-p12.1 (“MMVP1”, identified in 1999) [23], chromosome 11p15.4 (“MMVP2”, identified in 2003) [24] and chromosome 13q31.3-q32 (“MMVP3”, identified in 2005) [25]. Furthermore, an X-linked form of mitral valve prolapse was also identified and mapped to Xq28 in 1998 [26]. Of these loci, the MMVP2 locus mutation was identified as a mutation in the *DCHS1* gene [27], and the X-linked form of mitral valve prolapse mapped to Xq28 was identified as a mutation in the *FLNA* gene [28]. Duval et al. [29] identified PTPN12 as a specific binding partner for the FLNA protein. Mutations in *FLNA* impaired the activity of two PTPN12 substrates, which could be involved in the pathophysiology of FLNA-associated mitral valve prolapse. In 2017, the first autosomal recessive form of mitral valve prolapse was described and linked to the *PLD1* gene [30]. The most recent genetic cause of mitral valve prolapse was found in the *DZIP1* gene, a gene associated with ciliogenesis [31]. Altered cilia-dependent regulation of the extracellular matrix could be a potential underlying mechanism of mitral valve prolapse. However, in these studies, no specific distinction was made whether the mitral valve prolapse was due to FED or Barlow’s disease.

Recently, van Wijngaarden et al. [32] performed whole exome sequencing in 101 consecutive probands with Barlow’s disease or a positive family history for mitral valve abnormalities and investigated the genetic variants found in 522 genes associated with cardiac development and/or disease. Only one patient (1%) had a likely pathogenic variant in one of the known causative genes (*DCHS1*), but 11% of the probands had a likely pathogenic variant in several cardiomyopathy genes (*DSP*, *HCN4*, *MYH6* and *TTN*). This study suggested for the first time that, particularly in Barlow’s disease, cardiomyopathy genes may also be associated with mitral valve prolapse. However, further studies, including functional tests, are needed to demonstrate the potential of these new candidate genes and find new ones to further understand the genetic background of mitral valve prolapse.

## 5. Clinical Characteristics

Clinical presentation is usually different between FED and Barlow’s disease (Table 1). At the moment of diagnosis, patients with FED are mostly older than 60 years and present with relatively acute symptoms, mostly in relation to chordal rupture. In turn, patients with Barlow’s disease normally present between 40 and 60 years of age, frequently have a long history of a murmur found as a coincidental finding during physical examination, and are often asymptomatic [3,17]. On physical examination, the murmur of patients with Barlow’s disease is a high-pitched, late, systolic murmur with a mid-to-late systolic click, whereas patients with FED have a harsh, holosystolic murmur [3].

### 5.1. Arrhythmias

Mitral valve prolapse is known to be associated with ventricular arrhythmias, such as complex premature ventricular contractions, nonsustained ventricular tachycardia and, in a small percentage of patients, i.e., up to 3%, sudden cardiac death [33]. Several risk factors are identified, such as female sex, younger age, inferior T-wave inversion on electrocardiogram, frequent premature ventricular contractions or nonsustained ventricular tachycardia assessed with Holter monitoring [34,35] and nonsyndromic, bileaflet prolapse, such as in Barlow’s disease [36,37,38], without significant regurgitation. However, mitral valve leaflet flail with acute onset of severe mitral regurgitation, such as in FED, is also associated with sudden cardiac death [39]. More recently, mitral annular abnormalities, such as mitral annular disjunction (MAD) and mitral annular dilatation [38,40], together with myocardial fibrosis at the level of the papillary muscles [41], were also associated with the arrhythmic phenotype of mitral valve prolapse. In addition, genetic and extravalvular factors may also contribute to the arrhythmogenic phenotype [42]. Although several risk factors, as mentioned above, are identified, there is no general consensus on which parameters are predictors of ventricular arrhythmias.

### 5.2. Concomitant Valvular Abnormalities

Mitral valve prolapse may also present with concomitant other valvular abnormalities. Initial studies suggested a higher prevalence of MR due to mitral valve prolapse in patients with bicuspid aortic valve [43,44], but more recently, Padang et al. [45] reported in 30,689 patients that the prevalence of mitral valve prolapse is similar between bicuspid and tricuspid aortic valve patients (2.7% vs. 3.4%).

Tricuspid valve pathology, such as redundant leaflet tissue and prolapse, seems to also be frequently associated with mitral valve prolapse, although not often resulting in significant tricuspid valve regurgitation [46]. A study by Hirasawa et al. [47] recently compared tricuspid valve geometry using three-dimensional (3D) transesophageal echocardiography between FED and Barlow’s disease. They observed that patients with Barlow’s disease had larger tricuspid valve annulus areas, billowing height and billowing areas compared to patients with FED and, importantly, that the occurrence of significant residual tricuspid regurgitation after concomitant tricuspid annuloplasty was greater in patients with Barlow’s disease than in patients with FED. Therefore, they hypothesized that degenerative changes of the mitral valve in Barlow’s disease might occur in a similar manner on the tricuspid valve.

## 6. Echocardiographic Characteristics

Standard two-dimensional (2D) echocardiography plays an essential role in the diagnosis of mitral valve prolapse, the quantification of MR severity and its impact on LV performance. Transthoracic echocardiography can identify a mitral valve prolapse, but a detailed analysis of mitral valve lesions to define FED or Barlow’s disease usually requires transesophageal echocardiography. This precise morphological evaluation of the mitral valve is necessary mainly in the case of severe MR with surgical indication in order to plan the operation and predict the likelihood of a successful mitral valve repair.

**Figure 2:**
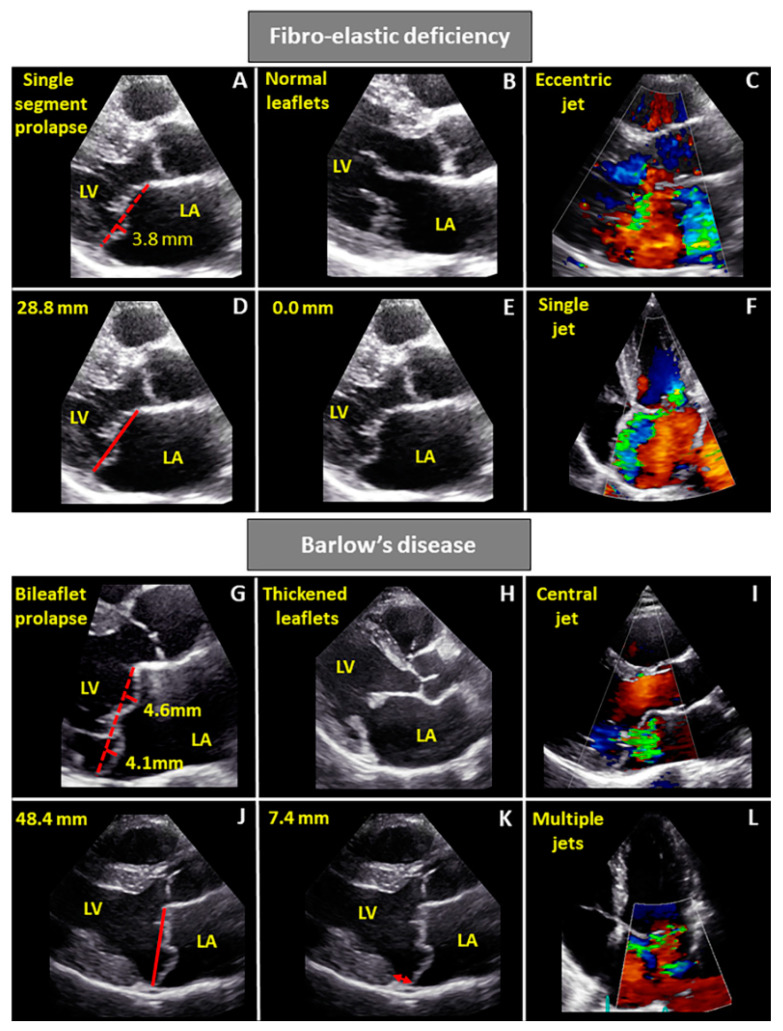
Transthoracic echocardiography: Differences between fibroelastic deficiency and Barlow’s disease. Transthoracic echocardiography images (parasternal long-axis view (**A**–**E**,**G**–**K**) and four-chamber view (**F**,**L**)) presenting the differences between FED and Barlow’s disease. Panels **A** and **G** displays the difference in prolapsing segment, including the prolapse height. Patients with FED have a single segment prolapse compared to the bileaflet prolapse of Barlow’s disease patients. As displayed in panels **B** and **H**, patients with Barlow’s disease have thick leaflets compared to the thin leaflets in FED. Panels **D** and **J** demonstrate that the mitral valve annulus diameter is larger in Barlow’s disease compared to FED, where the annulus is within the normal range. Patients with Barlow’s disease often have MAD, as presented in panel **K** with the red arrow, whereas this is rare in patients with FED (panel **E** showed no signs of MAD). Panels **C**, **F**, **I** and **L** show = mitral regurgitation in both patients, with patients with Barlow’s disease more often having multiple jets compared to the single jet in FED patients. FED = fibroelastic deficiency, LA = left atrium, LV = left ventricle and MAD = mitral annular disjunction.

Using echocardiography, a mitral valve prolapse is defined as an abnormal systolic leaflet movement into the left atrium, ≥2 mm beyond the saddle-shaped annular level measured in the parasternal long-axis [48] (Figure 2). In FED, echocardiography usually shows a single, prolapsing segment, mostly with a visible ruptured chorda, whereas the rest of the valve has normal or thin leaflets without redundant tissue. In most cases, the middle scallop of the posterior leaflet (P2) is involved, but in principle, any valve segment, including anterior or posterior commissures, could be involved [3]. The associated MR jet is usually holosystolic and eccentric, directed to the opposite side of the prolapsing segment [3,49,50]. Finally, in these patients, mitral annular diameter is often within normal range, but in some cases mild annular dilatation can occur [17,50]. In turn, Barlow’s disease is characterized on echocardiography by a diffuse, redundant leaflet tissue, with bileaflet prolapse or prolapse of multiple segments of the same leaflet. Valve leaflets are also often thickened (>3 mm) as measured in diastole using the M-mode [3,50]. Chordae are also frequently thickened and chordal elongation is more common than chordal rupture [17,46]. Severe annular dilatation is another common feature of Barlow’s disease, with frequent calcifications, especially in the advanced stage of the disease (Figure 2, Table 1) [3].

Another mitral annular abnormality often described in the presence of mitral valve prolapse is mitral annular disjunction (MAD), described as a separation between the left atrial wall at the level of the mitral valve junction with the LV free wall [51]. Mantegazza et al. [52] recently evaluated the occurrence of MAD in a large cohort of patients (*n* = 979) with Barlow’s disease or FED who underwent surgery. The authors showed that the prevalence of MAD was higher in patients with Barlow’s disease compared to FED (22% vs. 6%), although the maximal MAD distance did not significantly differ between the two phenotypes. In another recent study, Hiemstra et al. [53] assessed the evolution of mitral valve abnormalities over time in patients with Barlow’s disease. Severe MAD (defined as ≥5 mm) was present in 38% of the patients with Barlow’s disease, whereas it was not observed in the healthy controls. Moreover, no significant changes in the occurrence of severe MAD were observed over time in Barlow’s patients, but the MAD distance slightly increased during follow-up.

### 6.1. LV Function Assessment

As mentioned above, echocardiography plays an essential role also in the assessment of the impact of MR on the LV performance. Currently, LV size and ejection fraction are important criteria for surgical indication in patients with severe primary MR [54,55]. However, more recently novel parameters such as global longitudinal strain (GLS) were proposed to improve assessment of LV performance. Hiemstra et al. [56] evaluated whether LV GLS was associated with long-term survival after mitral valve surgery, and compared patients with Barlow’s disease and FED. This study revealed that patients with FED had more impaired LV GLS preoperatively compared to patients with Barlow’s disease (20% vs. 22%), although no significant differences in LV dimension, volume or ejection fraction were observed. Overall, patients with a more impaired LV GLS (based on the mean LV GLS) showed worse survival.

### 6.2. 3D Echocardiography

**Figure 3:**
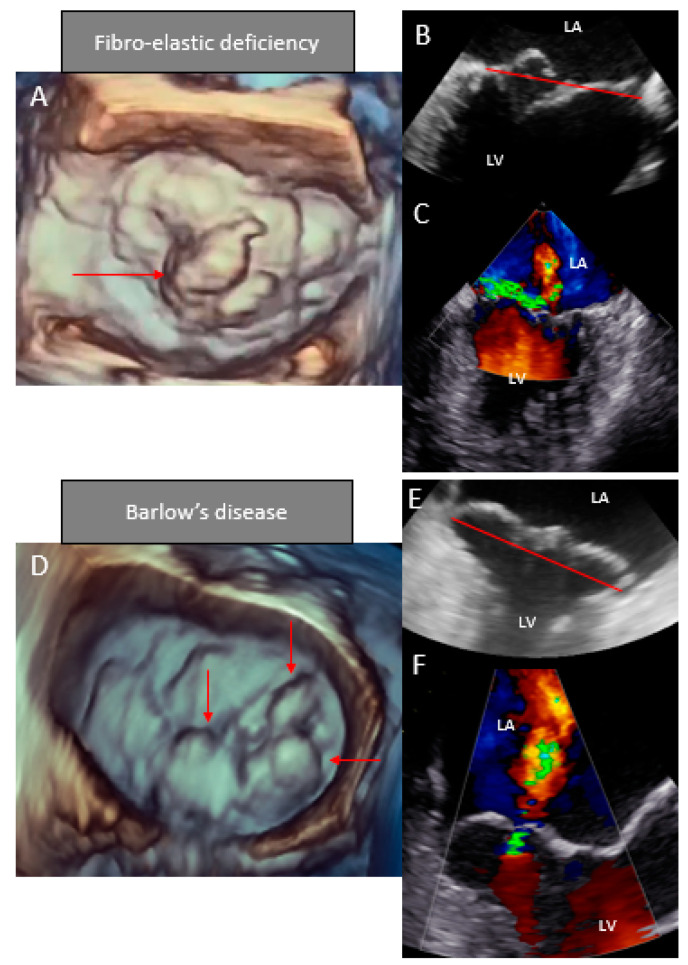
Two-dimensional (color) and three-dimensional transesophageal echocardiographic differences between fibroelastic deficiency and Barlow’s disease. Transesophageal echocardiography including 3D images for a patient with fibroelastic deficiency (**A**–**C**) and a patient with Barlow’s disease (**D**–**F**). Panels **A** and **D** display the 3D transesophageal images, showing the detailed anatomy of the valve with a single prolapsing segment in panel **A** and a multiscallop, bileaflet prolapse in panel **D** (red arrows). Panels **B** and E show the 2D view with the mitral valve annulus plane (red line); the patient with fibroelastic deficiency presented a single segment prolapse and the patient with Barlow’s disease a bileaflet prolapse. Panels **C** and **F** show the color-Doppler images with mitral regurgitation. LA = left atrium and LV = left ventricle.

3D echocardiography, particularly transesophageal, was shown to allow a more accurate assessment of the mitral valve morphology and function compared to 2D echocardiography, providing detailed descriptions of the valve lesions and therefore helping in characterizing FED versus Barlow’s disease [50]. 3D echocardiography can also provide proper quantification of valve geometry and dynamics (Figure 3) [57]. In 2011, Chandra et al. [49] proposed an algorithm to differentiate between FED and Barlow’s disease based on mitral valve geometry parameters using 3D transesophageal echocardiography. They found that billowing height of 1.0 mm could differentiate between normal valves and degenerative mitral valve disease, and that a billowing volume of 1.15 mL could distinguish between Barlow’s disease and FED. Other studies evaluated the annular dynamics comparing FED and Barlow’s disease. Clavel et al. [58] included 31 patients with FED and 18 patients with Barlow’s disease and found an almost normal annular dynamics in FED patients, whereas patients with Barlow’s disease showed significant annular enlargement and flattening in (end)systole, reflecting the typical systolic outward motion of the mitral valve annulus observed in Barlow’s disease, previously called “curling”. Similarly, van Wijngaarden et al. [59] evaluated the mitral annulus dynamics with 3D transesophageal echocardiography in 52 patients with FED and 40 patients with Barlow’s disease and assessed the saddle-shape of the mitral annulus with the annulus height to commissural width ratio. Patients with Barlow’s disease showed the largest annular dilatation (as commissural width and annulus area) and more pronounced configurations of the saddle-shape, as demonstrated by an increase in the annulus height to commissural width ratio at early systole and a decrease in ratio at late systole. Also, using 3D echocardiography, Viani et al. [60] classified degenerative MR into five categories: FED, FED with myxomatous leaflet changes, Barlow forme fruste, Barlow’s disease and Barlow’s disease with mitral annular disjunction. The results showed that an increase in phenotype severity was associated with mitral annular enlargement and flattening and that prolapse height and volume increased along with more severe phenotypes.

## 7. Cardiac Magnetic Resonance Imaging (CMR)

CMR is another imaging modality that can be used to better assess patients with mitral valve prolapse, and therefore with FED or Barlow’s disease. CMR can provide a 3D analysis of mitral valve morphology and dynamics, as in the study by Sturla et al. [61], where the authors used a CMR-dedicated framework and showed, similarly to the echocardiographic findings, that the mitral annulus is larger and has a rounder shape in patients with Barlow’s disease compared to FED. Furthermore, they showed that patients with Barlow’s disease have higher prolapse height and volume. CMR can also be used to detect MAD. Essayagh et al. [62] assessed the prevalence of MAD in patients with either myxomatous mitral valve disease (Barlow’s disease phenotype) or FED. MAD was more frequently diagnosed in patients with myxomatous mitral valve disease than in FED (60% vs. 14%).

**Figure 4:**
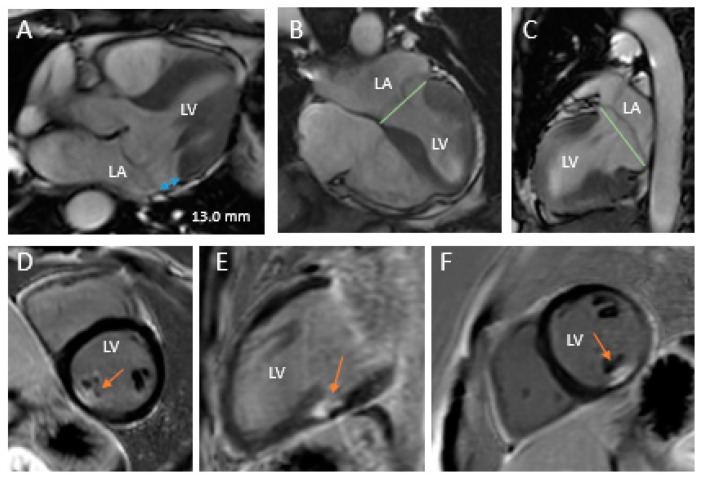
CMR examples of patients with Barlow’s disease. CMR images of two patients with Barlow’s disease. Panels (**A**–**C**) represent Patient 1 with Barlow’s disease, showing mitral annular disjunction (blue arrow) and a clear bileaflet prolapse. Panels (**D**–**F**) represent Patient 2 with late gadolinium enhancement at the level of the papillary muscle (orange arrow). CMR = cardiac magnetic resonance imaging, LA = left atrium and LV = left ventricle.

CMR is currently considered the gold standard for the assessment of LV volume, mass and ejection fraction [50,63], and represents a very accurate approach to assess MR severity when echocardiographic findings are conflicting [64]. Importantly, CMR has the unique ability of myocardial tissue characterization, and late gadolinium enhancement (LGE) can be used to demonstrate areas of myocardial fibrosis (Figure 4) [41], which are of particular interest in patients with bileaflet prolapse (mostly Barlow’s disease) and ventricular arrhythmias [65]. However, studies making a clear comparison between the CMR results in FED and Barlow’s disease are missing.

## 8. Surgical Approach

Currently, the indication for surgery is based on the severity of mitral regurgitation and the presence of symptoms, LV dilatation and dysfunction, atrial fibrillation or pulmonary hypertension [54,55]. Mitral valve repair is preferred over mitral valve replacement and the likelihood of repair therefore also influences the indication and timing of surgery. The feasibility of mitral valve repair largely depends on the complexity of mitral valve alterations, and the surgical approach is often different between FED and Barlow’s disease according to the mitral valve lesions. Usually an annuloplasty is performed using a ring to stabilize the annulus in both type of patients. In addition, different surgical repair techniques, such as annular plication, leaflet resection, leaflet sliding and neochord implantation, were developed with the goal of correcting any leaflet prolapse, as well as addressing excessive leaflet tissue in height and/or in width and preventing systolic anterior motion of the mitral valve [66]. Inspection of the valve during surgery reveals differences between FED and Barlow’s disease, and while in most FED cases neochordae implantation is sufficient, Barlow’s valves often require different resecting techniques. Some studies hypothesize that patients with Barlow’s disease (bileaflet prolapse) have a higher recurrence rate of severe mitral regurgitation due to the higher complexity of the disease and the repair [67]. Another study performed by Tomsic et al. [68] explored the early and late outcomes of mitral valve repair in patients with Barlow’s disease and, despite the complex valve abnormalities in patients with Barlow’s disease, the early and late outcomes were excellent, with low rates of recurrence of mitral regurgitation after 10 years follow-up.

## 9. Outcome

The natural course of degenerative mitral valve disease, including FED and Barlow’s disease, varies significantly and is influenced by several factors such as mitral regurgitant volume, LV function and pulmonary pressures [69]. Asymptomatic patients with only mild mitral regurgitation often remain stable for several years, and only a small proportion of the patients develop severe mitral regurgitation [69].

Complications such as endocarditis and stroke are described in the literature. Several studies found that the risk of infective endocarditis is increased in the presence of mitral valve prolapse. Katan et al. [70] included more than 800 patients with mitral valve prolapse and revealed that the relative risk of infective endocarditis in patients with mitral valve prolapse was 8% compared to the general population adjusted for age and sex. Moreover, patients with MR grade 2 or higher showed a higher incidence of infective endocarditis as compared to patients with less than grade 2 MR, and patients with flail mitral leaflet also showed an increased incidence of infective endocarditis compared to those without. However, current guidelines do not recommend antibiotic prophylaxis in these patients [54,55].

Some studies demonstrated an association between stroke and mitral valve prolapse [71]. Avierinos et al. [72] studied mitral valve patients in sinus rhythm without a history cerebral vascular events and reported that patients with mitral valve prolapse had an increased risk of stroke, although independent determinants of this outcome were atrial fibrillation and older age.

Although mitral valve repair for FED and Barlow’s disease have been performed for many years, robust data on outcomes after surgery are sparse. A recent study of Hiemstra et al. [56] assessed the difference in all-cause mortality between patients with FED and Barlow’s disease undergoing mitral valve repair, and showed that patients with Barlow’s disease had a similar prognosis as patients with FED after correcting for age.

## 10. Conclusions

Barlow’s disease and FED present with specific clinical and morphological features, with consequent differences in diagnosis and patient management. However, patients may share characteristics of both phenotypes and a clear distinction cannot always be made. Furthermore, the differences in the histological alterations of the leaflets and chordae of these patients remain unclarified and the exact pathophysiological mechanisms are not identified, leaving the question regarding whether Barlow’s disease and FED are part of a single degenerative mitral valve disease spectrum or two separate diseases unsolved.

## Figures and Tables

**Table 1:** Summary of the main differences between fibroelastic deficiency and Barlow’s disease.

	Fibroelastic Deficiency	Barlow’s Disease
**Clinical Characteristics**		
Age of onset	Older (>60 years)	Young (<60 years)
History	No history of murmur	Usually a long history of murmur
Duration of the disease	Months	Years to decades
Auscultation	Holosystolic murmur	Midsystolic click and late-systolic murmur
**Echocardiographic Characteristics/Surgical Inspection and Approach**		
Leaflets	Single segment (usually posterior) prolapse (flail) due to chordal rupture Thickened leaflet tissue (when present) is limited to the level of the prolapsing segment Thin/normal leaflet tissue in non-prolapsing segments	Diffuse excessive valve tissue with multiple segments, bi-leaflet prolapse Thickened leaflets
Annulus	Normal of moderate annular dilatation No calcifications	Severe annular dilatation Calcifications could be present Mitral annular disjunction Systolic outward motion during systole (curling)
Chordae	Chordal rupture of the involved segment	Elongated or ruptured Thickened and/or calcified
Repair approach	Respect tissue (annuloplasty and neochord implantation)	Resect tissue (annuloplasty, resection and sliding, neochord implantation)
